# Kitchen fine particulate matter (PM_2.5_) concentrations from biomass fuel use in rural households of Northwest Ethiopia

**DOI:** 10.3389/fpubh.2023.1241977

**Published:** 2023-10-17

**Authors:** Habtamu Demelash Enyew, Abebe Beyene Hailu, Seid Tiku Mereta

**Affiliations:** ^1^Department of Public Health, College of Health Sciences, Debre Tabor University, Gondar, Ethiopia; ^2^Department of Environmental Health Science and Technology, Institution of Health, Jimma University, Jimma, Ethiopia

**Keywords:** biomass fuel, kitchen concentration, fine particle, cooking, Ethiopia

## Abstract

**Background:**

Combustion of solid biomass fuels using traditional stoves which is the daily routine for 3 billion people emits various air pollutants including fine particulate matter which is one of the widely recognized risk factors for various cardiorespiratory and other health problems. But, there is only limited evidences of kitchen PM_2.5_ concentrations in rural Ethiopia.

**Objective:**

This study is aimed to estimate the 24-h average kitchen area concentrations of PM_2.5_ and to identify associated factors in rural households of northwest Ethiopia.

**Method:**

The average kitchen area PM_2.5_ concentrations were measured using a low-cost light-scattering Particle and Temperature Sensor Plus (PATS+) for a 24-h sampling period. Data from the PATS+ was downloaded in electronic form for further analysis. Other characteristics were collected using face-to-face interviews. Independent sample t-test and one-way analysis of variance were used to test differences in PM_2.5_ concentrations between and among various characteristics, respectively.

**Result:**

Mixed fuels were the most common cooking biomass fuel. The 24-h average kitchen PM_2.5_ concentrations was estimated to be 405 μg/m^3^, ranging from 52 to 965 μg/m^3^. The average concentrations were 639 vs. 336 μg/m^3^ (*p* < 0.001) in the thatched and corrugated iron sheet roof kitchens, respectively. The average concentration was also higher among mixed fuel users at 493 vs. 347 μg/m^3^ (*p* = 0.042) compared with firewood users and 493 vs. 233 μg/m^3^ (*p* = 0.007) as compared with crop residue fuel users. Statistically significant differences were also observed across starter fuel types 613 vs. 343 μg/m^3^ (*p* = 0.016) for kerosene vs. dried leaves and Injera baking events 523 vs. 343 μg/m^3^ (*p* < 0.001) for baked vs. not baked events.

**Conclusion:**

The average kitchen PM_2.5_ concentrations in the study area exceeded the world health organization indoor air quality guideline value of 15 μg/m^3^ which can put pregnant women at greater risk and contribute to poor pregnancy outcomes. Thatched roof kitchen, mixed cooking fuel, kerosene fire starter, and Injera baking events were positively associated with high-level average kitchen PM_2.5._ concentration_._ Simple cost-effective interventions like the use of chimney-fitted improved stoves and sensitizing women about factors that aggravate kitchen PM_2.5_ concentrations could reduce kitchen PM _2.5_ levels in the future.

## Introduction

Every day, nearly 3 billion people rely on solid biomass fuels (wood, dung, plant leaves, and charcoal) to cook their foods and to provide heat and light (1–3). Burning of these solid biomass fuels with open fires or inefficient stoves results in large amounts of health-damaging pollutants including a multitude of complex particulate matter and carbon monoxide (4–6) that exceed world health organization (WHO) air quality guidelines (24-h mean PM_2.5_ concentrations of 15 μg/m^3^) (7). Based on WHO report, in regions where solid biomass fuels are widely used, average levels of PM_2.5_ were very high in kitchens 972 μg/m^3^ and for personal exposure of women 267 μg/m^3^ (8). In Africa, especially in the east, west, central, and southern parts of the continent, an estimated three-fourths of the population relies on solid biomass fuels for cooking and is exposed to high concentrations of harmful pollutants at home every day (3, 9).

In Ethiopia, more than 95% of the population used solid biomass fuels for cooking and were exposed to kitchen smoke which is typical for low-income countries (10, 11). Evidences from rural Ethiopia showed that women, girls, and children at early age were exposed to extremely high levels of PM_2.5_ (12–14). Previous studies also reported 24-h average particulate matter concentrations of 818 ug/m^3^ in slum areas of Addis Ababa, 1,297 ug/m^3^ in three regions (Amhara, Oromia, and South Nation Nationalities and People) of Ethiopia, 772 μg/m^3^ in Wolaita Sodo town and 410 ug/m^3^ in Butajira town (13–16) all exceeded 24-h WHO safety level (17). As previously reported, these differences in concentrations may be due to differences in fuel and kitchen types, measuring devices, sampling seasons, and cooking patterns within households (13, 18–20).

Epidemiological studies are also increasingly showing that exposure to high levels of indoor air pollution from biomass fuel use kills millions and is a major contributor to global climate change (4–6). Household air pollution (HAP) contributed to more than 3.2 million annual premature deaths and 91.5 million disability-adjusted life years (DALYs) worldwide with a clear geographical variation where the majority of the burden is found in southeast Asia and sub-Saharan Africa (21–23). In 2019, air pollution was responsible for 1.1 million deaths across Africa, with more than half of those fatalities associated with household pollutants (24). Pneumonia and stroke are the leading causes of premature death due to HAP (3, 22, 23). About 400,000 children under 5 years old die each year as a result of HAP, primarily in sub-Saharan Africa and Asia (25).

In addition to detrimental cardiovascular effects, growing evidence shows potential perinatal risks associated with solid biomass burning (26–28). Adverse pregnancy outcomes such as low birth weight (LBW), pre-term birth (PTB), intrauterine growth restriction, and post-neonatal infant mortality are associated with biomass fuel smoke exposure (29). Fetuses are the most vulnerable stage to air pollution due to susceptibility at early ages (30, 31). In 2019, more than 100,000 deaths and 11.3 million DALYs related to preterm birth worldwide (66% in western sub-Saharan Africa and south Asia) were caused by excess PM_2.5,_ of which nearly two-thirds of them were attributable to household particulate matters PM_2.5_ (32).

According to the local burden of disease estimate in Ethiopia, exposure to HAP from solid biomass fuel use was the second highest risk factor for child pneumonia deaths next to child malnutrition (33). The available local epidemiological studies have reported strong correlations between elevated PM_2.5_ levels and acute respiratory infections (ARIs) among under-five children (16, 34–36). In Adama (southeast Ethiopia), HAP causes premature death and a significant number of DALYs due to biomass fuel use among women (37). Other existing evidences in Ethiopia revealed that the prevalence of acute respiratory infection including pneumonia among under-five children in households using solid biomass fuel remains high, ranging from 8 to 30 percent (34, 38–40).

Research on kitchen area concentration of particulate matter is limited in Ethiopia. Even the available evidences reported different results due to differences in the technologies used in the measurements, the sampling period, the study area (urban vs. rural), the season of measurements (dry vs. wet), the fuel and kitchen types, housing conditions, and other characteristics. Therefore, measuring local kitchen PM_2.5_ concentrations and understanding different factors that influence kitchen particle concentration can inform measures to maximize the effectiveness of various interventions.

## Methods and materials

### Study setting

This study was conducted in a low-income rural community of the south Gondar zone, northwest Ethiopia as part of the ongoing stove intervention study. Pregnant women were recruited from six kebeles (the smallest administrative unit) of the Guna–Tana integrated field research and development center catchment area. The field research center was established in 2013 by Debre Tabor University to integrate education, research, and community services. It is located 650 km away from the capital city of Ethiopia, Addis Ababa, toward northwest Ethiopia and 105 km far away from the capital city of Amhara regional state, Bahir Dar. Solid biomass fuel is exclusively a household energy source for cooking with traditional three-stone stoves in the study area. Kebeles in the two ecological zones (cold and temperate) were included to represent a diversity of characteristics expected to influence kitchen concentration of particulate matter including altitude, cooking practices, fuel types, and socioeconomic conditions. Tobacco smoking is uncommon and vehicle emission is almost negligible in the study community.

### Study design and population

A cross-sectional data was analyzed using the baseline measurements from an ongoing improved stove randomized controlled field trial study^1^ to estimate PM_2.5_ concentrations in kitchens of pregnant women cooking with solid biomass fuel in traditional stoves. The study participants who fulfilled the eligibility criteria were randomly selected and recruited from households in the stove trial project. To be eligible and participate in this study, a pregnant woman must meet the following inclusion criteria: Aged 18–38 years, being the primary cook of the household, in her first or second-trimester gestation (gestational age ≤ 24 weeks), exclusively using the traditional biomass-fueled stove or locally modified mud stove and having enclosed cooking area separated from or attached to the main house. But, pregnant women who had the plan to move permanently outside the study area in the next 12 months and who are engaged in local alcohol production activities were excluded from the study.

### Sample size

The number of households with eligible pregnant women for kitchen PM_2.5_ concentration measurement was determined based on standard conventional power calculations in the HAP intervention studies (41). These standard conventions include achieving a statistical power of 0.80, a value of p of 5% in two-tailed tests, and detecting a 64% HAP reduction due to an improved stove from a previous study (14). But, a reliable Ethiopia-based estimate of the coefficient of variation in HAP reduction was not available before our study to compute the minimum sample size. Therefore, a conservative COV estimate of 0.7 (41) was used which gave a minimum sample size of 43 households in each arm (a total of 86 households with pregnant women). Hence, all the baseline data collected from the upcoming stove trail study were analyzed for 86 randomly selected households.

### Variable definitions and measurements

#### Kitchen

In this study, the kitchen is used to indicate all enclosed cooking spaces separated from or attached to the main house in rural households.

#### Kitchen types

**Figure 1:**
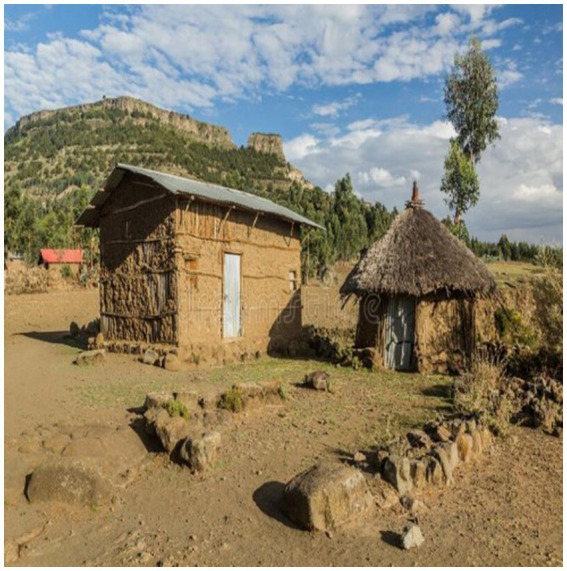
Small thatched roof kitchen near the main house.

**Figure 2:**
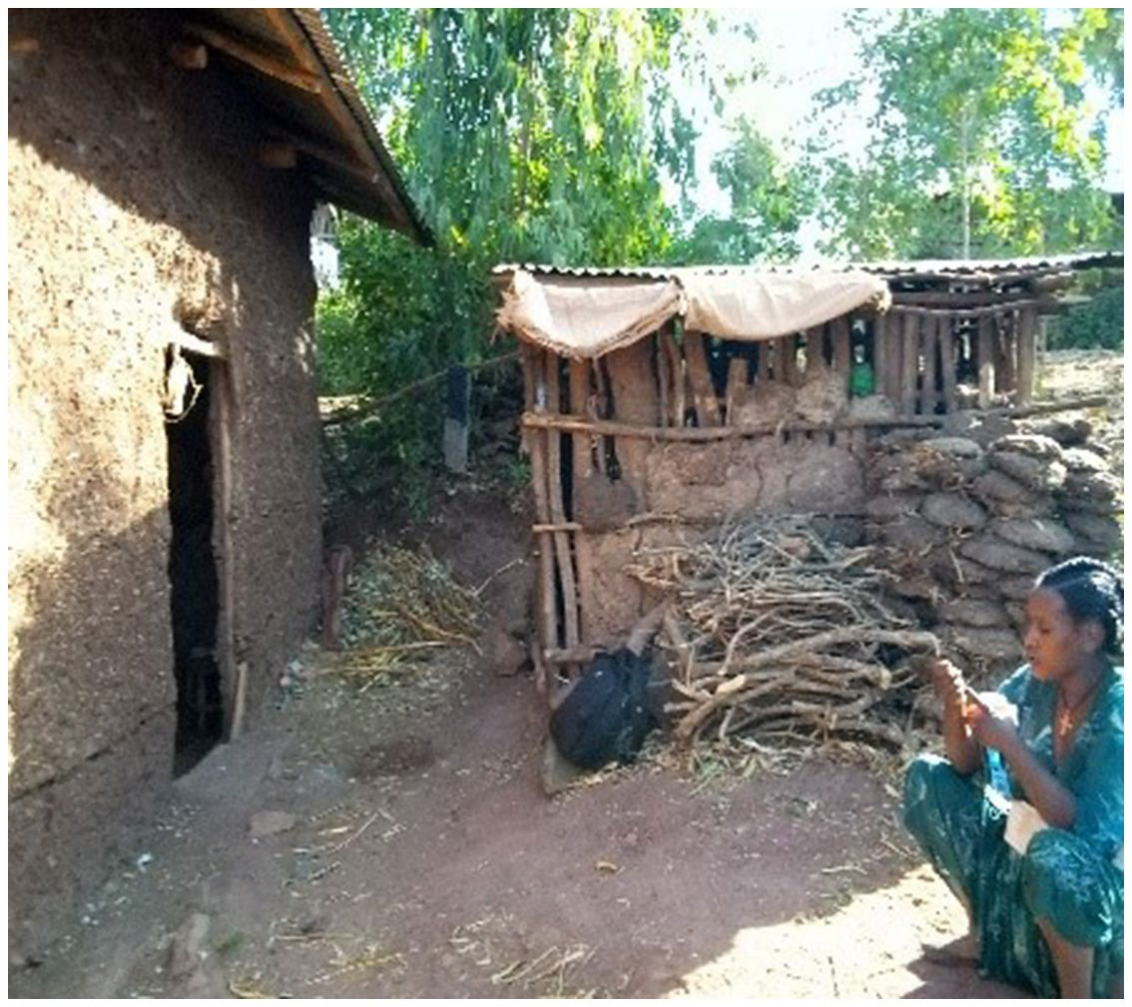
Small corrugated iron sheet roof kitchen near the main house.

There were two main kitchen types included in this study. The first one is a small thatched-roof kitchen near the main house. This type of kitchen had low-lying ceilings and very tightly enclosed walls resulting in the accumulation of dense biomass smoke during meal cooking due to the lack of an outlet at the highest part of the roof (Figure 1). The second kitchen is the small congrugated iron sheet(CIS) roof-enclosed kitchen with outlets between the wall and the roof for smoke removal (Figure 2).

#### PM_2.5_ concentrations

It is the daily average concentrations of PM_2.5_ calculated for the 24-h sampling period. Continuous PM_2.5_ measurements were done using PATS+ following standard protocol. In this study, the device logged particle concentration with a logging interval of 1 min.

#### Biomass fuel

Any plant or animal matter which when burned provide heat or light. The type of cooking fuel was re-categorized into three classes; (a) firewood (b) cow dung (c) agricultural residue and (d) mixed fuels (using two or more biomass fuels together).

#### Primary biomass fuel

It is the first fuel choice that is usually cheap and easily available in villages. It’s the primary practical option for rural households.

#### Family size

The total number of individuals permanently living in the household was assessed by recording all individuals (male, female, under-five children) and further categorized as (a) less than five individuals and (b) greater than or equal to five individuals. This classification was based on the average household size in Ethiopia reported by the Ethiopian Demography and health survey of 2016 (10).

### Data collection procedures

#### Survey

All relevant baseline data were collected as part of an ongoing randomized controlled trial study. Face-to-face interviews using structured and pretested questionnaires and observational checklists were conducted by trained first-degree environmental health professionals in the local language (Amharic). The key data were collected on economic status (using the list of assets owned by the households), housing characteristics (floor, wall, roof, number of rooms, windows, and doors), kitchen characteristics (size, presence of windows, and location), fuel types, frequency of cooking, and frequency of Injera baking. Injera is the staple food in Ethiopia which is a flatbread-like pancake prepared from a tiny grain called Teff. Baking Injera is very energy-intensive to cook which uses approximately 50% of the energy consumed in the household (42). We also collected updated information on fuels used and the time activity pattern of the day during the particle measurement phase.

### Particulate matter (PM_2.5_) measurements

In this study, kitchen PM_2.5_ concentrations were measured in 86 households using Particle and Temperature Sensor Plus (PATS+) which is a light-scattering particle sensor developed by Berkeley Air Monitoring Group, California. PATS+ is quite popular in this field as it is easy to transport and required less place to install. It had an internal power supply for 80 h of continuous measurement after being completely charged and provided data in a minute interval of time (43, 44). The device had a lower particulate matter detection limit of 10 μg/m^3^ and an upper particulate matter detection limit of 50,000 μg/m^3^ with a logging particle concentration interval of 1 min. Previous field validation tests have shown that PATS+ relates well to gravimetric PM_2.5_ estimates in laboratory settings (*R*^2^ = 0.97) and in rural biomass-using households (*R*^2^ = 0.74) (43).

PATS+ was calibrated using gravimetric filters co-located in a previous study conducted in Ethiopia. Based on the regression result, an adjustment factor was estimated to be 0.8065 (14). But, for this particular study, it was not possible to calibrate the instrument specifically for local particulate matter due to the harsh sampling environments. Instead, we conducted side-by-side inter-comparison tests between PATS+ and DylosDC1700 air monitor devices in a real setting in 11 kitchens following standardized experimental procedures. The result confirmed good data comparability across PATS+ devices (Pearson correlation coefficients: 0.75 to 0.86).

A 24-h continuous kitchen air monitoring was carried out covering all Ethiopian main meals of the day (breakfast, lunch, and dinner) during the study period. Then, for each household, average concentrations were calculated as the means of these minute-by-minute average concentrations for each household with data on a sufficient number of hours (more than 20 h). In each household, monitoring for PM_2.5_ started in the morning at around 8:30 am. We used the morning to the morning as starting and ending points of a sampling day. A total of 4 PATS+ were rotated through households during this study.

### Instrument placement in the kitchens

**Figure 3:**
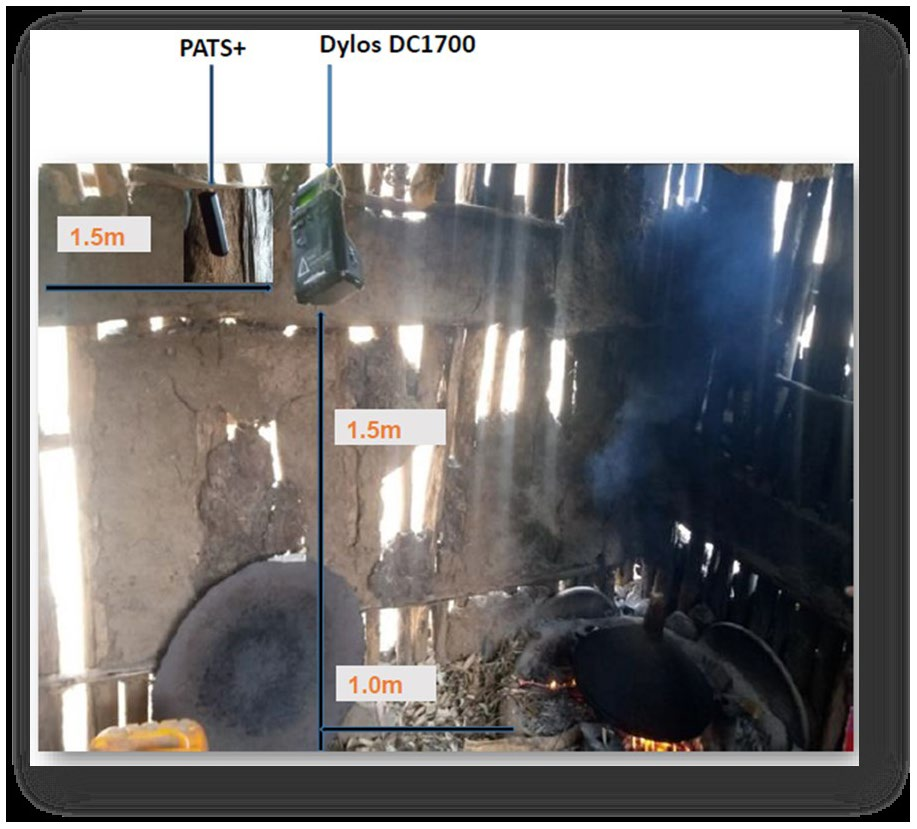
Placement position of particulate matter monitoring devices in the kitchen area.

The air monitoring devices were placed in the main kitchen at least 1 m away from the edge of the stove (to prevent from damaging as the devices cannot tolerate extreme temperatures and to represent the general cooking area), at a height of 1.5 m above the floor (the approximate breathing height of standing women), 1.5 m away from doors, windows, and other openings horizontally (to minimize ambient air entering the room) (45), and at a safe location to minimize the risk of interrupting normal household activities or being disturbed (Figure 3). The air monitors were attached to a wall or suspended from the ceiling and run for 24 h to consider households’ typical daily cooking activities. In addition to measuring mean PM_2.5_ concentrations, the PATS+ monitors also measured humidity and temperature.

### Data quality

Field workers were trained in the use of the sampling equipment (PATS+ and Dylos DC1700), and a detailed manual with pictorial aids developed by the Berkeley air monitoring group (46) was used to assist them. They instructed to follow the standard operating procedures for installing indoor air pollution instruments in a home (45) in gathering kitchen air samples. To ensure that each 24-h period was representative (capturing a typical number of cooking events), measurements of PM_2.5_ concentrations were removed from analysis when the total sampling time was shorter than 20 h.

In a previous study, PATS+ has been validated against gravimetric samples in Ethiopian settings, with the resulting strong linear correlation (*r*^2^ > 0.80) (14). We have also conducted side-by-side inter-comparison tests between PATS+ and DylosDC1700 air monitor devices in a real setting in 11 kitchens following standardized experimental procedures which yield comparable data across PATS+ devices (Pearson correlation coefficients: 0.75 to 0.86). All PATS+ were zeroed in a plastic bag for 10 min before and after deployment in the kitchens. Though readings of optical air monitors are significantly affected by relative humidity levels usually at >80%, the relative humidity recorded in this study area ranged from 53 to 61% and would be unlikely to affect readings by more than 5% as reported from previous literature (47).

### Data analysis

Data from the PATS+ air monitoring devices were downloaded in electronic form using the Platform for Integrated Cook Stove Assessment (PICA) software to the computer with CSV format Excel spreadsheets and text files. Paper-based data on the socioeconomics and demographic characteristics including housing conditions, kitchen types, fuel types, and cooking behavior were entered into SPSS software. Before formal statistical analyzes, simple tabulations and diagrams were constructed to gain a good understanding of the data and to identify gross outliers. Then, descriptive statistics including frequencies and percentages for categorical variables, and mean and standard deviations for continuous variables were calculated and presented using tables and graphs. In addition, we examined the pick hours at which the pollutant concentration in the kitchen measures high.

One-way analysis of variance (ANOVA) within 95% limits of a confidence interval, and value of *p* <0.05 was used to test differences in PM_2.5_ concentrations among different characteristics with multiple levels. Tukey’s Honest Significant Difference (HSD) test was done following ANOVA, to assess the significance of differences between pairs of groups. An independent sample t-test was used to check for differences in PM_2.5_ concentrations between two different characteristics at a significant level of 5%. Data were analyzed using the statistical package for social science (SPSS) version 24.0 software and Microsoft Excel for better graphical visuals.

### Ethical approval

This study was approved by the institutional review boards of Jimma University with ethical clearance provided (Ref No: IHRPGD/538/2021) to conduct the study. Information about the purpose of the study and potential study outcomes were provided to all participants. All participants were asked to give consent for participation before the commencing of the data collection. As a significant proportion of this population was illiterate, verbal informed consent was received from all participating households. Official letters of cooperation were given to the south Gondar zone health department and respective district health offices and permission to conduct the study was obtained. The right of the respondent to withdraw from the interview or not to participate was respected. During air pollution monitoring sessions, field staff received permission from participants to place air pollution monitoring devices in their kitchens. Devices chosen for pollution have no risk for participants.

## Results

### Household characteristics

In this study, a total of 86 households (HHs) with eligible pregnant women were approached for kitchen PM_2.5_ concentration measurement. However, air monitoring data from 3 HHs were discarded due to the following reasons; (a) in one HH, an air monitoring device (PATS+) was taken from the kitchen to the main house to prevent it from theft, (b) in another HH, an air monitoring device was covered with the cloth to prevent it from damaging by children and (c) data from the third HH was discarded due to short sampling period (18 h).

All participants were Amhara by ethnicity, Orthodox Christian, and most of them were married. They were living on an earthen floor, wood/mud wall, and corrugated iron sheet (CIS) roof house which is typical in the study area. The mean age of the respondents was 28.7 (SD ± 5.34) years. In this study, there were an average of 4.5 (SD ±1.4) individuals permanently living in the household.

### Kitchen characteristics

All households included in this study had a one-roomed separate kitchen with earthen floors and without windows. Nearly, three-fourths of participants had congregated iron sheet (CIS) roofed kitchens 64 (76.7%) and the rest 19 (23.3%) cooked in thatched roofed kitchens near the main house. When cooking, the kitchen doors of all participants’ kitchens opened partially or completely. The thatched roof kitchens have no sufficient opening to vent out cooking smoke, making pregnant women more vulnerable. Whereas the kitchens with CIS roofs, though there were no formal ventilations, there were openings between the wall and the ceiling which provided informal ventilation and reduces smoke exposure.

### Cooking practices

All participants lived in households where cooking was regularly practiced. Mixed fuels (mainly wood with dung) were the most common fuels used by 34 (40.7%) of the respondents followed by firewood where 27 (32.6%) of the interviewed pregnant women used to cook their food. Nearly three-fourths of the participants 62 (74.7%) used additional fuel to start the kitchen fire, from whom 22 (35.5%) used dried plant leaves and 19(30.6%) used agricultural straws. All participants were baking Injera at least twice per week and other meals daily (average cooking time = 2.8 (SD 0.92) hours/ day) for an average of 5 (SD 1.4) individuals during the study period (Table 1).

**Table 1:** Cooking related characteristics and distribution of kitchen PM_2.5_ concentration in rural households of north-west Ethiopia (*n* = 83).

Characteristics	Number	Mean (SD) of PM_2.5_ (ug/m^3^)
Age group, in years		
18–24	19	481 [155]
25–31	35	435 [205]
32–38	28	321 [253]
Kitchen roof material		
Corrugated metal roof	64	336 [182]
Thatched roof	19	639 [181]
Types of fuel used during the study period		
Wood	28	358 [190]
Dung	14	414 [204]
Crop residues	9	231 [167]
Mixed fuels	32	493 [233]
Baking *Ijera* during the measurement period		
Yes	29	523 [209]
No	54	343 [202]
Family size		
≥ 5 individuals	42	427 [229]
<5 individuals	41	384 [212]
Number of meals cooked per day		
Twice	10	377 [160]
Three times	28	392 [211]
Four and more times	45	420[240]
Kitchen size		
< 15m^3^	27	485 [255]
≥15 m^3^	56	367 [193]
Use another fuel to start the fire		
Yes	62	407 [223]
No	20	402 [222]
Types of fuel used to start the fire (*n* = 62)		
Leaves	22	343 [190]
Straw	19	372 [245]
Paper	13	443 [182]
Kerosene	8	613 [224]
Number of meals cooked per day		
One meal	10	377 [160]
Two meals	28	392 [211]
Three and more meals	45	420 [240]
Opening between the kitchen wall and roof		
Yes	15	316 [226]
No	67	429 [215]

### Kitchen PM_2.5_ concentration

The average daily sampling time per household was 22.7 h with a range of 21 to 24 h. Because, some of the participating women faced unexpected social issues like funerals, health problems, and other family issues that enforced them to go far away from their residences. In this case, they have to lock their kitchen and the installed air monitors have to be uninstalled. Since we planned to consider measurements undertaken for more than 20 h, we excluded one measurement due to the short sampling period (18 h). The average temperature was 20.4°C, while the average humidity was 57% for the cooking area.

**Figure 4:**
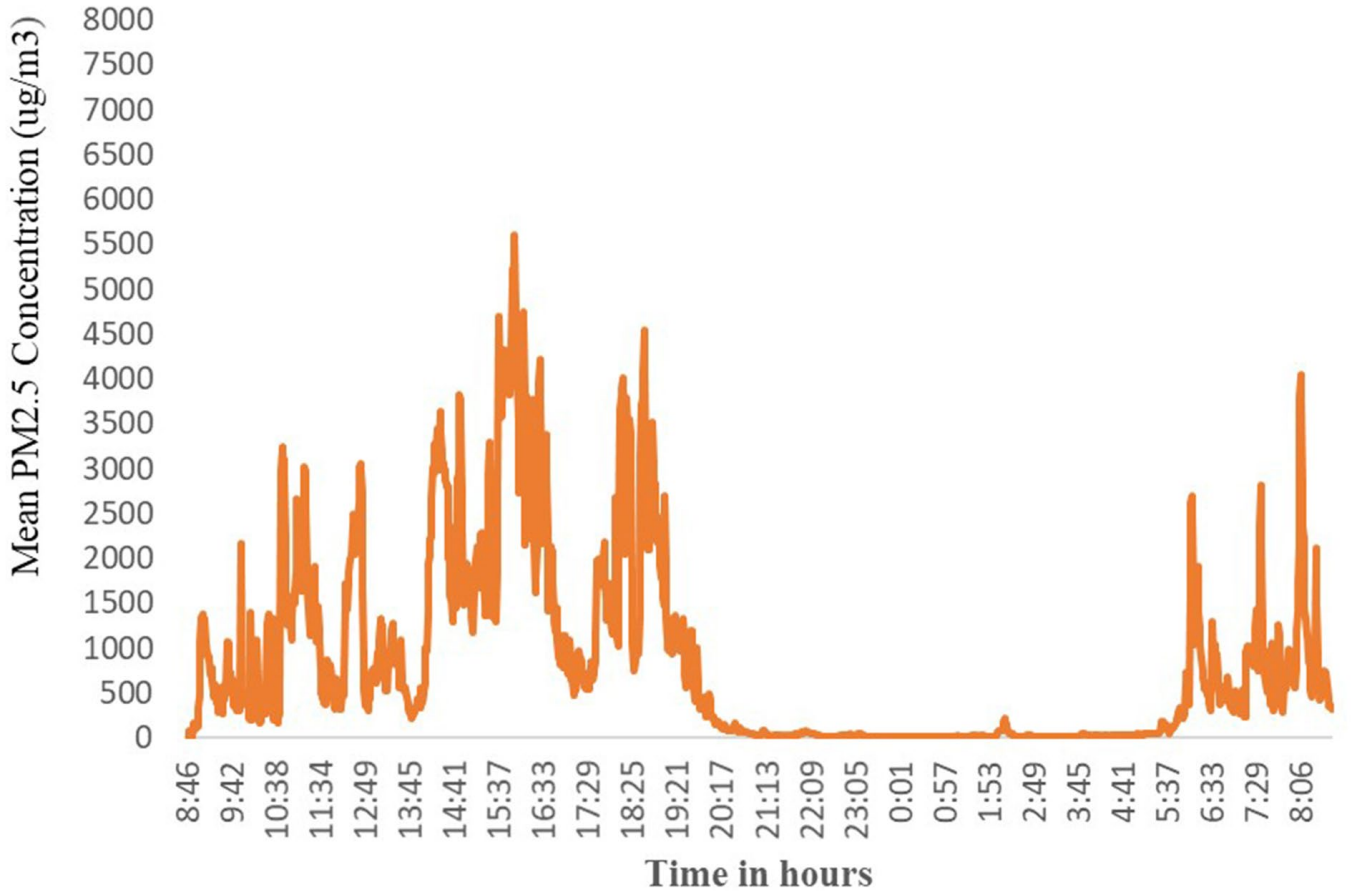
The distribution of kitchen area hourly average concentrations of PM_2.5_ (μg/m^3^) by the time of day.

The average 24-h kitchen area PM_2.5_ concentrations were estimated to be 405 μg/m^3^ (SD 221 μg/m^3^) ranging from 52 to 965 μg/m^3^ and the median concentrations were less than the mean at 383 μg/m^3^. The continuous PM_2.5_ concentration profile consistently showed slight diurnal peaks reflecting morning and evening cooking periods and was lowest overnight when the stove was likely off (Figure 4).

### PM_2.5_ concentrations by kitchen characteristics

**Figure 5:**
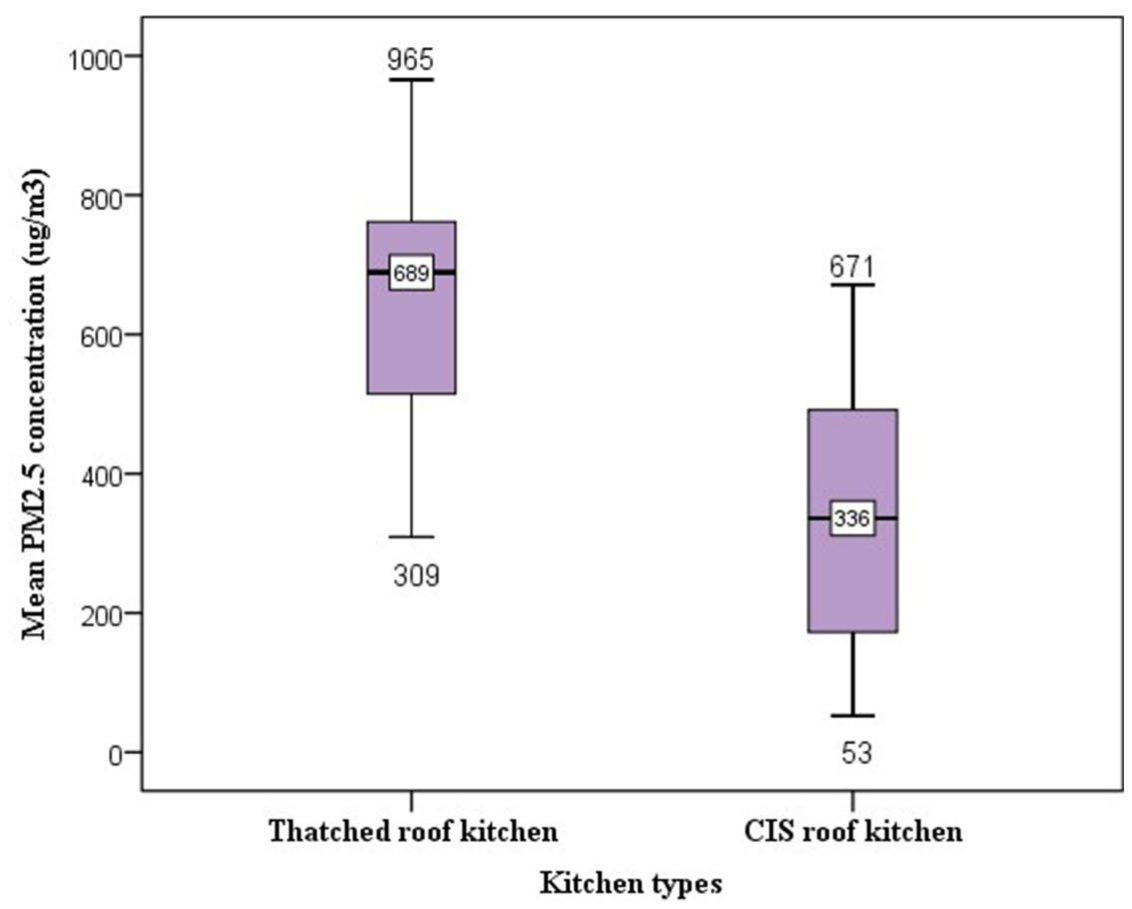
Box and whisker plots of 24- h kitchen PM_2.5_ concentrations by kitchen roof type. The ends of the box are at quartiles, so that the length of the box is the interquartile range (IQR). The median is marked by a line within the box. The two whiskers outside the box extend to the smallest and largest observations.

The presence of an enclosed kitchen was one of the criteria for the HHs to be included in the kitchen area PM_2.5_ concentration measurement. The average PM_2.5_ concentrations was highest in the kitchen with a thatched roof (639 μg/m^3^) with daily average concentration ranging from 309 to 965 μg/m^3^ as compared with CIS roofed kitchen at an average concentration of 337 μg/m^3^ with a range from 52 to 671 μg/m^3^ (Figure 5).

The difference in average PM_2.5_ concentrations is mainly due to a lack of outlet between the wall and the roof in the thatched roof kitchen where cooking smoke is trapped. Because the thatched roof kitchens had low-lying ceilings and very tightly enclosed walls resulting in the accumulation of dense biomass smoke during meal cooking. While the CIS roofed kitchen had many outlets at the highest part of the roof which served as smoke removal.

### PM_2.5_ concentrations by fuel types

**Figure 6:**
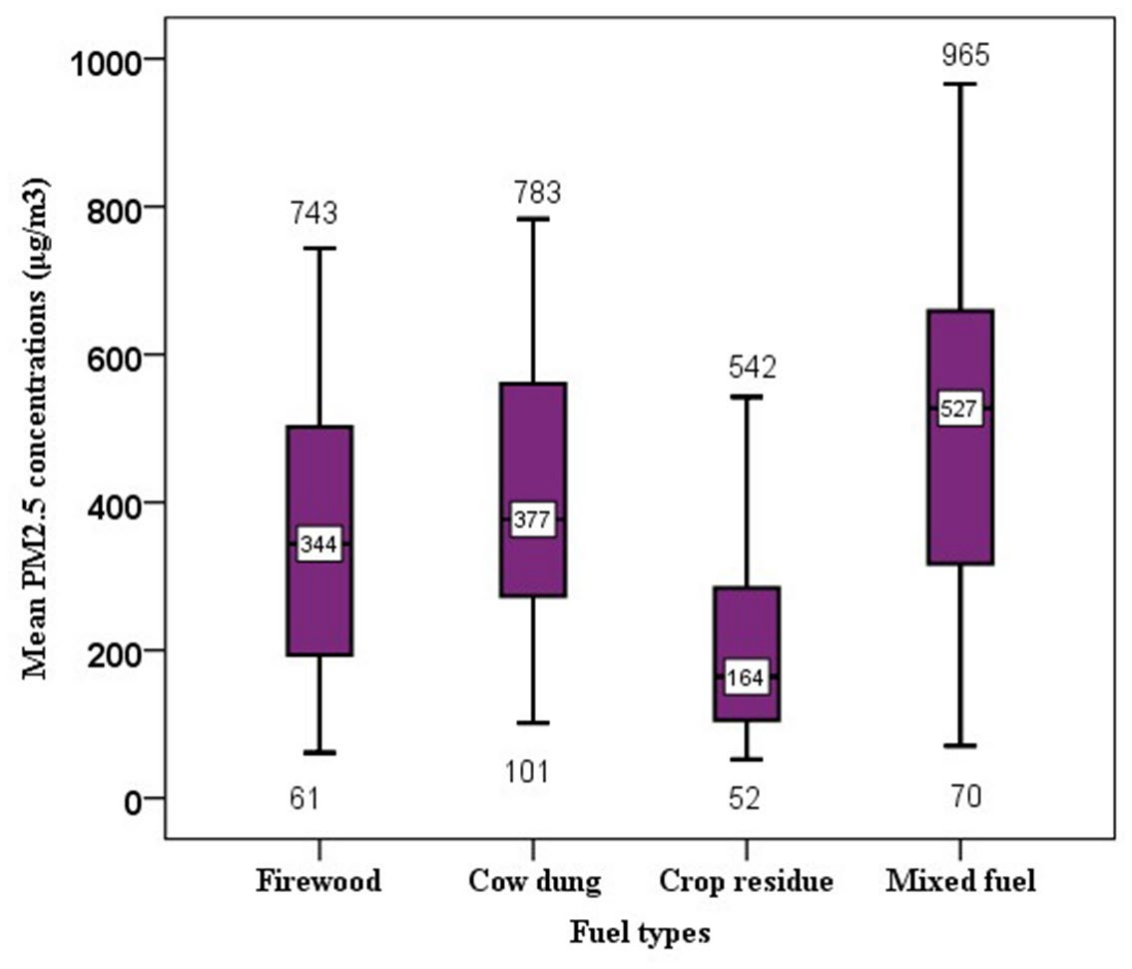
Box and whisker plots of 24- h kitchen PM_2.5_ concentrations by types of cooking fuel used during sampling period. The whole boxes represent the interquartile range; numbers on the horizontal line inside the box indicate median. The top and bottom whiskers are minimum and maximum values, respectively.

In this study, the average PM_2.5_ concentrations vary with different biomass fuel types used to cook the meal. Burning of mixed biomass fuel in the kitchen produces the highest average PM_2.5_ concentrations. In the kitchens where mixed fuel was used, the average PM_2.5_ concentrations were estimated to be 493 μg/m^3^ with a median of 527 μg/m^3^. The corresponding average concentration in kitchens with cow dung fuel was estimated to be 414 μg/m^3^. For firewood cooking fuel, the average particle concentration was 358 μg/m^3^ with a median of 344 μg/m^3^ and the least particle concentration was recorded among agricultural residue users at the PM_2.5_ concentrations of 231 μg/m^3^ (Figure 6).

**Figure 7:**
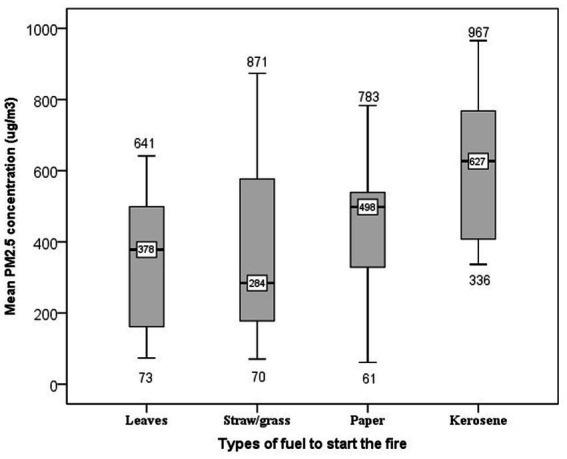
Box plot presenting the 24-h Kitchen area PM_2.5_ concentrations using different fuel types to start the fire in the kitchen. The whole boxes represent the interquartile range; numbers on the horizontal line inside the box indicate median. The top and bottom whiskers are minimum and maximum values, respectively.

In addition to cooking fuel, the use of additional starter fuel to initiate the wood fire affects the concentration of particles in the kitchen. Accordingly, in the kitchen where kerosene was used to start the fire, the average PM_2.5_ concentrations was 493 μg/m^3^ followed by 414 μg/m^3^ among straw/grass starter fuel users (Figure 7).

### Determinants of daily average kitchen concentrations of PM_2.5_

In addition to graphical visualization of raw relationships between different factors and average particle concentration, the model-based analysis provides a quantitative confirmation of important findings. As a result, we used the one-way analysis of variance (ANOVA) test to determine whether there is a significant difference in the mean concentration of PM_2.5_ by each of, the fuel types used to cook, fuel types used to start the fire, number of meals cooked per day and other variables with more than two groups.

An independent sampling t-test was also used to compare the average concentration of two different groups and check for significant differences between these average concentrations. All significance values of Levene’s test/statistics based on a comparison of the average concentration were greater than 0.05 indicating the requirement of homogeneity of variance has been met and the ANOVA and independent sample t-tests can be considered to be robust.

Accordingly, a statistically significant difference was observed in average PM_2.5_ concentrations between the thatched roof and CIS roof kitchens. The results indicated that cooking in a thatched roof kitchen emitted on average 639 μg/m^3^ (SD = 181) PM_2.5_ concentrations, compared with cooking in a CIS roofed kitchen which emitted an average concentration of 336 μg/m^3^ (SD = 182) PM_2.5._ This difference was statistically significant at 0.05 level (*t* = 6.37, *p* < 0.001). Using eta-square to examine the effect size, about 33.4% of the variation of PM_2.5_ concentrations could be explained by kitchen roof types.

Similarly, in the kitchen where *Injera* was baked the average concentration of PM_2.5_ was recorded to be 523 μg/m^3^ (SD = 209), compared with the kitchen where *Injera* was not baked which emitted an average of 343 μg/m^3^ (SD = 202) PM_2.5._ This difference was statistically significant at the 0.05 level (*t* = 3.81, p < 0.001). Based on the eta-square effect size estimate, only 15.2% of the variation could be explained by Injera baking events.

Regardless of the kitchen type, the result of one-way ANOVA showed a significant difference between fuel types used during the air monitoring period (firewood, cow dung, crop residue, and mixed fuel) and the average concentration of PM_2.5_ (*F* = 4.46, *p* = 0.006). A Tukey *post hoc* test showed that burning of mixed biomass fuel (mean = 493 ug/m^3^, SD = 233 ug/m^3^) emitted significantly high average PM_2.5_ concentrations than using both firewood (mean = 347 ug/m^3^, SD = 189 ug/m^3^) and agricultural residues (mean = 232 ug/m^3^, SD =167 ug/m^3^). But there is no significant difference in average kitchen PM_2.5_ concentrations among firewood, cow dung, and crop residue users.

Similarly, a statistically significant difference was observed between the types of fuel used to start the fire (dried leaves, straw/grass, and kerosene) and the average concentration of PM_2.5_ (*F* = 3.48, *p* = 0.021). Accordingly, a Tukey *post hoc* pairwise comparison test showed that the use of kerosene to start the fire (mean = 613 ug/m^3^, SD = 224 ug/m^3^) has significantly higher average PM_2.5_ concentrations than using dried plant leaves (mean = 343 ug/m^3^, SD = 190 ug/m^3^), straw/grass (mean = 372 ug/m^3^, SD = 245 ug/m^3^) and papers (mean = 443 ug/m^3^, SD = 182 ug/m^3^) to initiate the fire in the kitchen. Although 24-h average PM_2.5_ concentrations at different meal cooking frequencies and the presence of openings between the kitchen wall and roof differed, the pairwise comparison indicated that it is not statistically significant (*p* > 0.05; Table 2).

**Table 2:** Cooking practices and kitchen characteristics associated with average PM_2.5_ concentrations in rural households of north-west Ethiopia.

Characteristics	Average PM_2.5_ difference (ug/m^3^)	95% CI	*p*-value	Eta square
Kitchen types				
Thatched roof	Reference	–		33.4%
CIS roof	303	[260, 344]	<0.001	
Fuel types used				
Mixed fuel	Reference	–		14.5%
Firewood	146	[4, 288]	0.042	
Cow dung	63	[−107, 233]	0.766	
Crop residues	261	[56, 466]	0.007	
Fuel types used to start the fire				
Kerosene	Reference	–	–	15.3%
Dried leaves	269	[38, 501]	0.016	
Straw/grass	240	[5, 477]	0.044	
Papers	170	[−81, 422]	0.289	
Injera was baked				
Yes	Reference	–		15.2%
No	180	[86, 273]	<0.001	
Number of meals cooked per day				
One meal	Reference	–		
Two meals	–15	[−211, 181]	0.982	
Three and above meals	–43	[−229, 143]	0.846	
Opening between the wall and roof				
Yes	Reference	–		
No	−113	[−237,10]	0.072	

## Discussion

In the study area, solid biomass fuels are often used with inefficient and poorly vented cook stoves that result in a high concentration of toxic pollutants (13, 48, 49). In this study, mixed fuels (mainly firewood with cow dung) were the main type of fuel used for cooking. Similar studies reported biomass fuel as the main domestic energy source for rural Ethiopia (11, 50, 51). The study on fuel consumption patterns in India also revealed that the majority of households used solid biomass fuel (predominantly cow dung and wood) for cooking (52). However, this study’s findings are different from results reported in Uganda and Kenya where charcoal and firewood only were reported to be the most commonly used cooking fuels, respectively (53, 54). These differences in fuel preference could be due to accessibility, types of a meal cooked, the design of used stoves, local temperature, and other behavioral and environmental factors.

In this study, the 24-h average kitchen area PM_2.5_ concentration was estimated to be 405 μg/m^3^ which is 27 times higher than the safety limit of 15 μg/m^3^ recommended by the WHO 24-h mean air quality guideline and five times higher than the most flexible interim WHO target (IT-I) of 75 μg/m^3^ (7) indicating the severity of kitchen area PM_2.5_ levels in study rural households.

This estimated 24-h average kitchen area concentration of PM_2.5_ was comparable to what is observed from the results of other kitchen air pollution monitoring studies in Ethiopia. Studies conducted in southern Ethiopia using similar (13) and different (15) air monitor devices in the kitchen have reported comparable results of 410 μg/m^3^ and 413 μg/m^3^, respectively. Another published review report in Ethiopia also revealed 24-h average PM_2.5_ concentration of 477 μg/m^3^ (55). Another measurement of PM_2.5_ during a single Injera baking event in Northwest Ethiopia reported an average PM_2.5_ concentration of 855 μg/m^3^ (56).

Nearly similar results were reported from studies conducted in India where a 24-h average concentration of 468 μg/m^3^ was reported (57) and in Nepal with a 48-h average concentration of 417 ug/m^3^ (58). A relatively higher concentration was reported in Pakistan where the average PM_2.5_ concentration was 531 μg/m^3^ (59) and in four states in India, 24-h average kitchen PM_2.5_ concentrations of 600 μg/m^3^ were reported (60). The differences in kitchen particle concentration suggest possible differences in local cooking practices, types of a meal cooked, and fuel types used. These high concentrations of PM_2.5_ as reported both from this study and previously conducted research in the kitchens might be due to the inefficient burning of biomass fuels and inefficient dispersion of particles in the kitchen area.

Because of the differences in kitchen design, the kitchen area concentration of PM_2.5_ also varies (61). Based on the independent sampling t-test, we found higher kitchen PM_2.5_ concentrations in households with thatched roof kitchens compared to households with metal sheet roofed kitchens. This result is similar to research reports conducted in Nepal and Peru where having metal sheet roof kitchens showed some association with decreased PM_2.5_ concentrations compared to roofs made of thatched/grass/straw (62, 63). Research results from Punjab in India also revealed that the concentration of PM_2.5_ varies across different kitchen types (52). As was also evidenced by another study, having a thatched roof was positively associated with increased 24-h PM concentrations (64). The possible reason might be due to better and faster dilution and dispersion of the pollutant taking place in different openings (in the case of metal sheet roofed kitchens) as compared to the confined kitchen (most thatched/grass roofed kitchens).

In this study, we also found that kitchen PM_2.5_ concentrations varied with different fuel types used for cooking. Hence, burning of mixed biomass fuels (mainly firewood with cow dung) emitted average higher PM_2.5_ concentrations than using firewood or agricultural residues only. A similar study on the effect of the fuel type used for cooking in the household showed that women who cooked with dung cake had the highest exposures compared with those who cooked with crop residues and firewood, respectively, in Ethiopia (12). Similarly, the maximum PM_2.5_ emissions were reported from the burning of dung cakes followed by agricultural residues and mixed fuel (wood and dung) uses in India (52). Another study in Nepal reported that biomass fuel was the most significant source of PM_2.5_ followed by kerosene (62). But, in Uganda, women who used crop residues had higher exposures to PM_2.5_ compared to those using wood (12). Because, many characteristics, including heating value, moisture content, chemical composition, and the size and density of the fuel, affect the amount of particles released and these characteristics can vary from fuel to fuel (65).

It is also common practice to use additional fuel to start the wood fire in the kitchen. Dried plant leaves, paper, kerosene, and straw/grass were commonly used wood fire starter fuels in the study area. We also found high PM_2.5_ levels variability by starter fuel type. In the kitchen where kerosene was used to start the wood fire, the average PM_2.5_ concentrations were higher followed by straw/grass users. It is also evidenced that rural Indian women commonly used kerosene to start a fire in the kitchen (66). Though the epidemiological evidence is limited in this regard, paper, plastics, or kerosene are used to start the fire because they have low ignition temperatures which help to catch fire immediately and help the wood or the dung to reach its required ignition temperature.

Recognizing the public health impact of HAP from biomass fuel use and considering the use of biomass fuels in developing countries is likely to remain stable in the near future, WHO suggested several practical interventions for a clean cooking transition before widespread affordable access to electricity (67–69). The introduction of locally acceptable improved stoves, improved housing and ventilation design (replacing thatched roof kitchen with CIS roofed kitchen), and education and awareness-raising to support necessary changes in cultural habits related to cooking are some of the strategies for reducing exposure to household air pollution (17, 56, 70). There is an evidence that a chimney-fitted improved stove reduced wood smoke exposures and was associated with reduced low birth weight occurrence (71). But the most effective way to improve indoor air quality is the use of cleaner fuels, such as biogas, ethanol, and liquefied petroleum gas (9, 17, 72) and electric, wind, and solar are the cleanest option for health (1, 67, 73) however, transition to these fuels is not yet feasible for low-income countries.

## Conclusion

Rural households in the study area entirely depend on biomass fuel with traditional three-stone stoves for cooking which emits high levels of particulate matter that exceeded WHO guideline values. The reported kitchen PM_2.5_ concentrations in this study are sufficiently high to be a cause for public health concerns. Since the average PM_2.5_ concentrations were found to be highest in the thatched roof kitchens, replacing the kitchen’s roof with CIS to ensure that it allows air exchange during cooking times may be of benefit. Types of cooking fuel, types of fuel used for igniting the cooking fuel, and Injera baking events are also significantly associated with higher PM_2.5_ concentrations. Simple cost-effective interventions like the use of chimney-fitted improved stoves could also reduce kitchen PM _2.5_ levels in the future. This study may be used as a starting point for intervention studies employing quantification of PM_2.5_ levels and other parameters that has to be considered in reducing the PM_2.5_ levels. Our findings also highlight the need to create awareness of the effects of HAP exposure and to identify best practices for reducing exposure in the kitchen to reduce pollution levels.

### Potential limitations

Though seasonal variations were reported in previous studies with high concentrations recorded during the cold season (19, 62), the presence of this variation was not captured in this study. The PATS+ measures fine particles at concentrations ranging from 10 to 50,000 ug/m^3^ and performed well when tested against a gravimetric standard. Due to the harsh sampling environment, we were unable to validate our continuous monitoring against the gravimetric analysis of samples collected in parallel. Therefore, the absolute values of the PM_2.5_ measurements may not be fully accurate and should be interpreted with caution. Although households were randomly selected for air monitoring from those participating in the stove trail study, the latter were recruited based on inclusion criteria which may exclude relevant households. Finally, we did not measure ambient air pollution and therefore cannot account for the proportion of concentration from ambient PM_2.5_ sources.

## Data availability statement

The raw data supporting the conclusions of this article will be made available by the authors, without undue reservation.

## Ethics statement

The studies involving humans were approved by the institutional review boards of Jimma University. The studies were conducted in accordance with the local legislation and institutional requirements. The participants provided their written informed consent to participate in this study.

## Author contributions

HE and AH: conceptualization, designing of the methodology, recruitment, training of supervisors, and data collectors. SM and AH: formal data analysis, interpreting the result, writing draft manuscript, and editing. All authors contributed to the article and approved the submitted version.

## Footnotes

^1^https://pactr.samrc.ac.za/; Identifier: ACTR202111534227089.
